# MOTS-c modulates pancreatic islet function in rats and pigs in vitro

**DOI:** 10.1007/s00418-025-02391-4

**Published:** 2025-06-06

**Authors:** Jakub Bień, Ewa Pruszynska-Oszmalek, Pawel Kolodziejski, Natalia Leciejewska, Dawid Szczepankiewicz, Emilia Grzęda, Maciej Sassek

**Affiliations:** Department of Animal Physiology, Biochemistry and Biostructure, Poznan University of Life Sciences, Poznan, Poland

**Keywords:** MOTS-c, Pancreas, Pancreatic islets

## Abstract

MOTS-c is a promising regulator of metabolism and energy homeostasis. While its effects have been studied in cell lines, our team aimed to investigate its influence on more complex structures—specifically, isolated pancreatic islets. We used two animal models: the rat, which is commonly studied, and the pig, which shares greater physiological similarities with humans. This study assessed the expression and secretion of insulin and glucagon, the expression of their receptors, cell viability, and cell death following MOTS-c treatment of the islets. Additionally, we examined how MOTS-c secretion is affected by different incubation media, such as the presence of free fatty acids, pancreatic hormones, and different glucose concentrations. The results indicate that MOTS-c impacts pancreatic islet physiology by, for example, reducing insulin and glucagon secretion and enhancing cell viability. Notably, the effects differed between the two species, which may be attributed to anatomical differences in their pancreatic islets or structural variations in rat and pig MOTS-c. These facts may lead to the conclusion that if MOTS-c may be helpful in human medicine, the pig model should be considered another valuable choice.

## Introduction

The continuous development of biological sciences continues to uncover new peptides that regulate key physiological functions. With the development of innovative research methods, new discoveries and insights are constantly emerging. Seventy years ago, the structure of DNA was described (Watson and Crick 1953). Ten years later, mitochondrial DNA was identified (Nass and Nass 1963). In the early twenty-first century, the first mitochondrial-derived peptide, humanin, was discovered in Hashimoto’s laboratory (Hashimoto et al. 2001).

Nearly a decade ago, MOTS-c peptide (mitochondrial open reading frame of the 12S rRNA-c) was identified. It was first discovered in silico as a short open reading frame within the 12S rRNA gene of the mitochondrial genome. MOTS-c is highly conserved across multiple species and is expressed in various tissues and organs, including the heart, kidneys, and muscles (Lee et al. 2015). Initial studies demonstrated that MOTS-c plays a significant role in metabolism and insulin resistance (Lee et al. 2015). Since then, many additional studies have explored its functions (Mohtashami et al. 2022). For example, it has been shown to provide protection in lipopolysaccharide-induced septic cardiomyopathy (Wu et al. 2023) and to exert a potential therapeutic effect in postoperative acute lung injury by mitigating damage and suppressing ferroptosis caused by myocardial ischemia–reperfusion (Lu et al. 2023). Moreover, MOTS-c plasma levels appear to be associated with obesity, as reduced levels have been observed in men, adolescents, and children with obesity (Du et al. 2018; Luo et al. 2023).

However, the functions of this peptide are still undiscovered and undescribed. In particular, limited research has investigated the peptide’s role in the pancreas and pancreatic islets. It is known that MOTS-c exerts a protective effect on pancreatic islets in type 1 diabetes mellitus (Kong et al. 2021) and that it alleviates hyperglycemia and insulin resistance in gestational diabetes mellitus (Yin et al. 2022).

In our previous study (Bień et al. 2024), we examined the effects of MOTS-c on pancreatic cell metabolism using laboratory cell line models (INS-1E and αTC-1). Since the results were promising for further research, we decided to examine how MOTS-c changes pancreas metabolism using a more complex structure as a test object—isolated pancreatic islets obtained from rats and pigs. Both animals are widely used as model organisms in biomedical research (Ericsson et al. 2013), and they offer contrasting pancreatic islets. In rats, the pancreas is dispersed throughout the intestinal mesentery, and the islets exhibit a distinct organization, with β-cells located centrally and α-cells at the periphery. In contrast, pigs have a more compact pancreas, and their islets lack such clear segregation, resembling the islet structure of the human pancreas more closely (Hoang et al. 2014).

## Materials and methods

### Animal information

Male Wistar rats, each weighing approximately 300 g, were obtained from the Animal House of the Mossakowski Medical Research Institute, Polish Academy of Sciences. Piglets of the Złotnicka White pig, weighing approximately 7–8 kg, were acquired from the Swadzim Agricultural Experimental Farm at the Poznań University of Life Sciences. All experimental procedures were conducted in accordance with Polish law and the guidelines of the Local Ethical Commission for Animal Research.

The MOTS-c peptide used in the experiments was purchased from Novazym (Poznań, Poland). For experiments involving rat islets, the rat-specific MOTS-c amino acid sequence—MKRKEMGYIFFSQRTLRNPL—was used. For experiments involving pig islets, the human MOTS-c sequence—MRWQEMGYIFYPRKLR—was used, as the porcine MOTS-c sequence has not yet been identified. Given that pigs are physiologically closer to humans than rats, especially in pancreatic structure, we chose to use the human MOTS-c peptide in our pig-based experiments.

### Isolation of rat pancreatic islets

Male Wistar rats were decapitated and exsanguinated, after which the dissection procedures were carried out immediately. The abdominal cavity was opened, and clamps were placed on the duodenum near the pancreatic duct estuary. The pancreatic duct was then severed, and a cannula was inserted. A total of 13 ml of Hanks’ balanced salt solution (HBSS, 0.137 M NaCl, 5.37 mM KCl, 4.17 mM NaHCO_3_, 1.26 mM CaCl_2_, 0.84 mM MgSO_4_, 0.44 mM KH_2_PO_4_, and 0.34 mM Na_2_HPO_4_) containing 13 Wunsch units of Liberase TL (cat. no. 05401020001, Roche, Basel, Switzerland) was injected into the pancreas. The pancreas was excised, transferred to a Falcon tube, and incubated in a water bath at 37 °C for 11 min. Following incubation, the tube was shaken vigorously for 30 s. The enzymatic digestion was stopped by adding HBSS supplemented with 10% fetal bovine serum (FBS). The islets were washed and manually isolated under a binocular microscope (Delta Optical, Poznań, Poland), then transferred into Krebs Ringer buffer (KRB, 115 mM NaCl, 24 mM NaHCO_3_, 5 mM KCl, 1 mM MgCl_2_, 1 mM CaCl_2_, and 0.5% bovine serum albumin, BSA) containing 6 mM glucose. They were incubated at 37 °C with 5% CO_2_ for 1.5 h to allow regeneration. Approximately 50 islets were collected for RNA or protein extraction. For RNA isolation, islets were frozen in 500 µl of TRIzol (cat. no. 15596026, Thermo Fisher Scientific, Waltham, MA, USA); for protein extraction, they were frozen in 200 µl of radioimmunoprecipitation assay buffer (RIPA, cat. no. 20-188, Merck Millipore, Burlington, MA, USA). All samples were stored at − 80 °C until further processing.

### Isolation of pig pancreatic islets

Pancreatic islets were isolated from the Złotnicka White piglets via enzymatic digestion of the pancreas. The animals were killed by an overdose of anesthetics and sedatives administration (medetomidine 200 µg/kg of body weight and ketamine 40 mg/kg of body weight) and then exsanguinated by intracardiac puncture. The pancreas was excised and placed in HBSS. Approximately 2 g of pancreatic tissue was transferred into a Falcon tube containing 10 ml of HBSS and finely chopped with scissors. Collagenase P (cat. no. 11213873001, Roche, Basel, Switzerland) was then added to the solution. The tube was incubated in a water bath at 37 °C for 12 min, followed by vigorous shaking outside the bath for 1 min. Enzymatic digestion was stopped by adding 90 ml of HBSS supplemented with 10% FBS. The islets were washed and manually isolated under a binocular microscope (Delta Optical, Poznań, Poland), then transferred to KRB containing 6 mM glucose. They were incubated at 37 °C with 5% CO_2_ for 1.5 h to allow for regeneration. Approximately 50 islets were collected for RNA or protein isolation. For RNA extraction, islets were frozen in 500 µl of TRIzol (cat. no. 15596026, Thermo Fisher Scientific, Waltham, MA, USA); for protein extraction, they were frozen in 200 µl of RIPA (cat. no. 20-188, Merck Millipore, Burlington, MA, USA). Samples were stored at − 80 °C until further analysis.

### Protein isolation

Pancreatic islets were incubated for 24 h in a 12-well plate. Following incubation, the islets were collected using RIPA buffer supplemented with cOmplete protease inhibitor tablets (cat. no. 04693116001, Roche, Basel, Switzerland) for protein isolation. The lysate was mixed using a vortex and centrifuged (two times). The tubes were then placed on a thermomixer and shaken at 900 rpm at 4 °C for 10 min. The samples were then centrifuged for 10 min at 13,000 × *g*. The resulting supernatant was carefully transferred to new tubes. Protein concentrations were measured using the Pierce BCA Protein Assay (Thermo Fisher Scientific, Waltham, MA, USA), following the manufacturer’s protocol.

### Western blot

The Western blot procedure followed the protocol described in Bień et al. (2024). Briefly, equal amounts of protein (15 µg) were separated by 12% gel electrophoresis (SDS PAGE) and transferred onto a 0.2-µm PVDF membrane. The membrane was blocked using 3% BSA and incubated overnight with the primary antibody 1:1000. After the membrane was washed with tris-buffered saline with Tween-20 (TBST), it was incubated with a secondary antibody 1:5000 for 1.5 h. Secondary antibodies were conjugated with horseradish peroxidase and the signal detection method was chemiluminescence. The signal corresponding to the target protein was measured. Subsequently, the membrane was incubated overnight with anti-β-actin or antiGAPDH antibody 1:5000, washed with TBST, and incubated again with a secondary antibody 1:5000. The signal for the reference protein was visualized using Super Signal™ West Pico PLUS (Thermo Fisher Scientific, Waltham, MA, USA) and detected using the ChemiDoc MP Imaging system (Bio-Rad, Hercules, CA, USA).

The antibodies and blocking peptide used for Western blotting were as follows: antiMOTS-c, rabbit polyclonal (cat. no. MBS542112, MyBioSource, San Diego, CA, USA); blocking peptide (cat. no. MBS543991, MyBioSource, San Diego, CA, USA); anti-β-actin (cat. no. A1978, Sigma-Aldrich, St. Louis, MO, USA); antiGAPDH (cat. no. G8795, Sigma-Aldrich, St. Louis, MO, USA). The secondary antibodies used were as follows: antirabbit IgG (cat. no. 7074P2, Cell Signaling, Danvers, MA, USA) and antimouse IgG (cat. no. A2304, Sigma-Aldrich, St. Louis, MO, USA).

### Immunofluorescence

Rat and pig pancreases were dissected out and placed in Bouin’s reagent (150 ml picric acid, 50 ml formaldehyde, 10 ml acetic acid; Sigma-Aldrich, St. Louis, MO, USA) immediately. Subsequently, fixed pancreases were embedded in paraffin, and sliced in 5-µm sections using a microtome. Paraffin was removed by heating the slides at 60 °C for 45 min. The sections were then rehydrated through graded alcohol solutions with decreasing concentrations (100%, 85%, 70%, 60%, 50%, water). Antigen retrieval was performed by boiling the slides in citrate buffer (pH 6, 10 mM disodium citrate and 0.05% Tween-20) three times for 5 min each, then cooling at room temperature for 20 min. Afterward, the samples were rinsed with water for 5 min. The tissue sections on the slides were encircled using a Dako Pen to contain the reagents during incubation.

To reduce autofluorescence, the samples were incubated in 15 mM glycine solution for 5 min, washed in phosphate-buffered saline (PBS), incubated in 0.2% gelatin solution, and washed again with PBS. Primary antibodies were then applied to stain for MOTS-c 1:200 (with and without 100 µl of blocking peptide in a concentration 2.5 mg/ml), insulin 1:400, and glucagon 1:400 in PBS buffer with 0.2% gelatin. Sections were incubated with primary antibodies overnight. For MOTS-c staining with blocking peptide, the antibodies were preincubated with the peptide for 48 h before application. Following primary antibody incubation, all samples were washed and incubated for 1 h in PBS containing 0.2% BSA. The secondary antibody was added for 15 min also in a concentration of 1:400 in PBS buffer with 0.2% gelatin. Finally, cell nuclei were stained with 4′,6-diamidino-2-phenylindole (DAPI) for 1 min. Images were captured using a Leica DMI8 fluorescence microscope (camera Leica K5-14403549, 16 bits, 2048 pixels, software system LAS X, objective HC PL FLUOTAR L 20×/0.40 DRY, aperture 0.4).

The antibodies and blocking peptide used for immunofluorescence were as follows: antiMOTS-c, rabbit polyclonal (cat. no. MBS542112, MyBioSource, San Diego, CA, USA); blocking peptide (cat. no. MBS543991, MyBioSource, San Diego, CA, USA); anti-insulin, polyclonal guinea pig (cat. no. A0564, Agilent, Santa Clara, CA, USA); and antiglucagon, polyclonal guinea pig (cat. no. 4031-01F, Merck Millipore, Burlington, MA, USA). The secondary antibodies used were Alexa Fluor 488 goat antiguinea pig IgG (cat. no. A11073, Life Technologies, Carlsbad, CA, USA) and Cy3 Goat antirabbit IgG (cat. no. A10520, Life Technologies, Carlsbad, CA, USA).

### MOTS-c secretion and cell culture medium with free fatty acids

Pancreatic islets were placed in a 48-well plate, with five islets per well, and incubated for 1.5 h in Krebs buffer. The control group received buffer without any additives, while experimental groups received Krebs buffer supplemented with: (a) 200 µM of oleic, stearic, or palmitic acid; (b) insulin at concentrations of 1, 10, or 100 nM; (c) glucagon at concentrations of 1, 10, or 100 nM. Additionally, rat and pig pancreatic islets were incubated for 1.5 h in experimental media containing glucose at concentrations of 2, 6, or 16 mM. MOTS-c levels in the incubation medium were measured using rat and pig MOTS-c ELISA kits (SunRed, Shanghai, China), following the manufacturer’s instructions.

Before islets were incubated in the medium, warm, free fatty acids were added to the medium, resulting in a final concentration of 200 µM of each free fatty acid. Then, the experimental medium was incubated for 1.5 h at 37 °C to couple free fatty acids to BSA in the medium.

### Secretion of hormones

Rat and pig pancreatic islets were incubated for 1.5 h in a 48-well plate with varying concentrations of MOTS-c (1 nM, 10 nM, and 100 nM or alternatively 10 nM and 100 nM in Krebs Ringer buffer), along with a control group that received no MOTS-c. After incubation, hormone concentrations were measured using ELISA kits specific to each species. Insulin levels were assessed using the Rat Insulin (INS) ELISA Kit and the Pig Insulin (INS) ELISA Kit (SunRed, Shanghai, China), while glucagon levels were determined using the Rat Glucagon (GC) ELISA Kit and the Pig Glucagon (GC) ELISA Kit (all from SunRed, Shanghai, China). All measurements were conducted according to the protocols provided by the manufacturer.

### RNA extraction, reverse transcription, and PCR

Pancreatic islets were incubated for 24 h in 12-well plates. Following incubation, total RNA was extracted using TRIzol according to the manufacturer’s instructions and was measured by Implen NP80 NanoPhotometer (Implen, Munich, Germany). Reverse transcription was then performed using the High Output cDNA Reverse Transcription Kit (Applied Biosystems, Waltham, MA, USA), following the protocol provided in the kit manual.

Quantitative PCR was carried out using the HOT FIREPol EvaGreen qPCR Mix Plus (Solis BioDyne OÜ, Tartu, Estonia) on the QuantStudio™ 12K Flex System (Thermo Fisher Scientific, Waltham, MA, USA). The primer sequences (5ʹ–3ʹ) were as follows. Rat insulin F: CCAGTTGGTAGAGGGAGCAG, R: AGACCATCAGCAAGCAAGCGGTC; insulin receptor F: CAGAAAAACCTCTTCAGGCAAT, R: TTCAAGGGATCTTCGCTTTC; glucagon F: AAGATGGTTGTGAATGGTGAAA, R: TGAGATGAACACGATTCTCGAT; glucagon receptor F: TTCTGTTGCAGACCAGCTCA, R: GTGACCAGTGCCACCACA; GAPDH F: CTGCACCACCAACTGCTTAG, R: TGATGGCATGGACTGTGG; pig insulin F: GTGGCATCGTGGAGCAGT, R: CGGCCTAGTTGCAGTAGTTCTC; insulin receptor F: AACGCCAGGGACATCGTCAA, R: CTTTGGACACCACCCCCAGG; glucagon F: CATCAGCCACTGCACAAAAT, R: AGGGCACGTTTACCAGTGAC; glucagon receptor F: GTCACGAAGGCAAACACCAC, R: CTGCCCTGGTACCACAAAGT; TATA-box binding protein (TBP) F: TTGAGAACATCTACCCTATCC, R: CGTCCACAACACCACCATT.

The PCR reaction was carried out in a total volume of 10 µl, consisting of 5 µl of reagent mix, 3 µl of cDNA, and 2 µl of primer mix (final primer concentration of 2.5 µM). Cycling conditions included an initial denaturation at 95 °C for 10 min, followed by 40 cycles of 15 s at 95 °C (denaturation), 1 min at 61 °C (annealing), and 20 s at 72 °C (extension). Melt curve analysis was performed with the following settings: 95 °C for 15 s, 60 °C for 60 s, and 95 °C for 15 s.

### MTT

Pancreatic islets were incubated with different concentrations of MOTS-c for 24 h. Following incubation, a 0.05% MTT solution (Merck Millipore, Burlington, MA, USA) was added to the islets. The plate was then incubated for 20 min at 37 °C. Afterward, the medium was removed, and the islets were dissolved in 100 µl of dimethylsulfoxide. Absorbance was measured at a wavelength of 570 nm, with background correction performed at 650 nm, using the Synergy 2 microplate reader (Agilent, Santa Clara, CA, USA).

### Apoptosis

Pancreatic islets were incubated with MOTS-c for 24 h in 96-well plates. The level of apoptosis was assessed using the Cell Death Detection ELISA Plus kit (Roche, Basel, Switzerland), following the manufacturer’s protocol. Absorbance was measured at a wavelength of 405 nm, with background correction performed at 490 nm, using the Synergy 2 microplate reader (Agilent, Santa Clara, CA, USA).

### Statistical analysis

All analyses were carried out using GraphPad Prism 6.0 software (GraphPad Software, San Diego, CA, USA). The results are presented as the arithmetic mean ± SEM. The significance of differences was determined using one-way analysis of variance (ANOVA) with a Dunnett post hoc test, comparing the results with the control group. Additionally, a Tukey post hoc test was employed when comparing groups with each other (experiments with glucose). Statistical significance is denoted by * for *p* < 0.05 and ** for *p* < 0.01.

## Results

### antiMOTS-c antibody is specific to pig MOTS-c

**Fig. 1:**
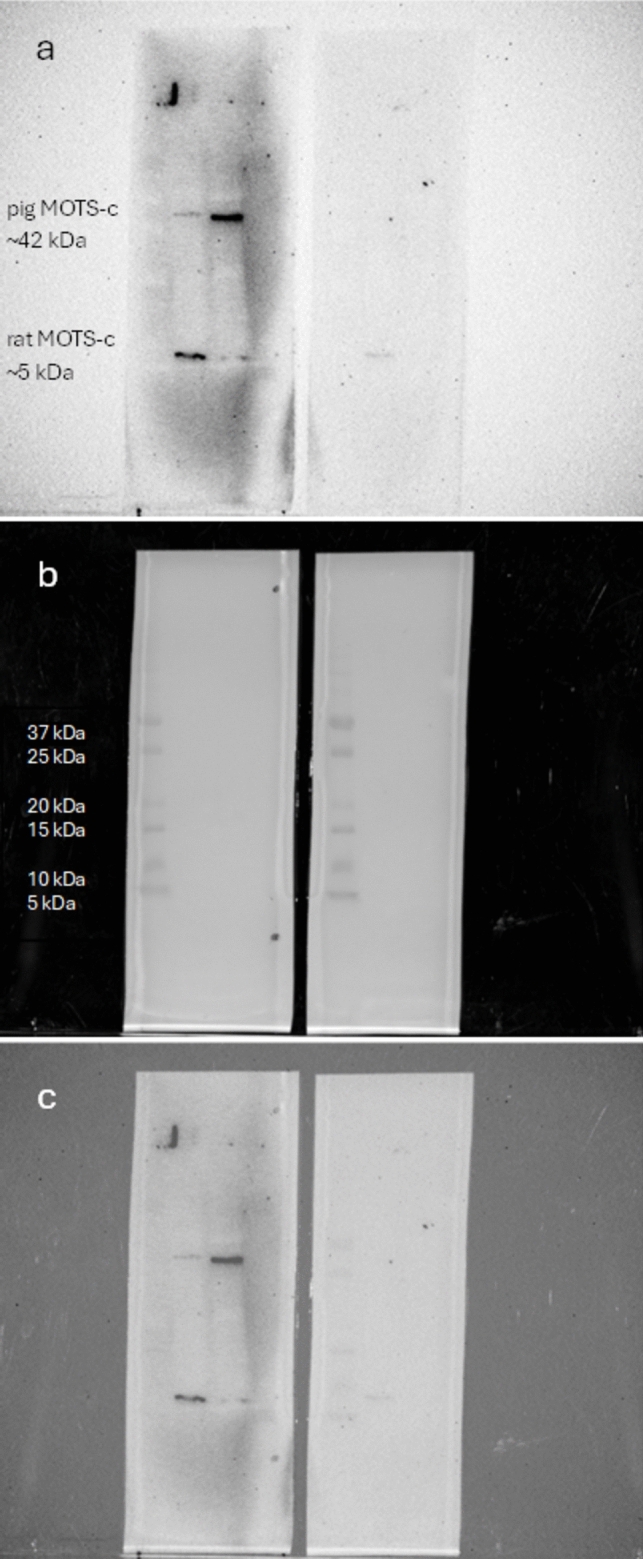
Confirmation that antiMOTS-c antibody is specific to pig MOTS-c. **a** Chemiluminescence, **b** visible light, **c** combination of** a** and** b**. Left membrane: left track, rat pancreas; right track, pig pancreas. There is a visible difference between the sizes of the MOTS-c protein in each species: right membrane, left track, rat pancreas; right track, pig pancreas. The molecular weight of rat MOTS-c is approximately 5 kDa, porcine MOTS-c is approximately 42 kDa. On the right membrane, an antibody blocked with MOTS-c peptide was used. The extinguishing of the signals from rats and pigs can be observed

To confirm the specificity of the antiMOTS-c antibody, Western blot analysis was performed using pancreatic tissue from rats and pigs (Fig. 1). Unfortunately, we do not have access to other validation methods related to genetics methods or mass spectrometry. On the membrane incubated with antiMOTS-c antibody, there was one clear signal in a sample from the rat pancreas and one clear signal in the sample from the pig pancreas. These signals disappeared when the antibody was preincubated with the MOTS-c blocking peptide dedicated to this antibody, confirming antibody specificity. Interestingly, MOTS-c signals were detected at different molecular weights, with pig MOTS-c appearing at a higher position than rat MOTS-c. This suggests differences in the amino acid sequence and/or tertiary structure between species, possibly due to the formation of distinct oligomeric complexes in pigs. Because the molecular weight of pig MOTS-c overlaps with that of β-actin (used as a reference protein), GAPDH was used instead as the control in Western blots involving pig tissue.

### MOTS-c is present in rat and pig pancreatic islets

**Fig. 2:**
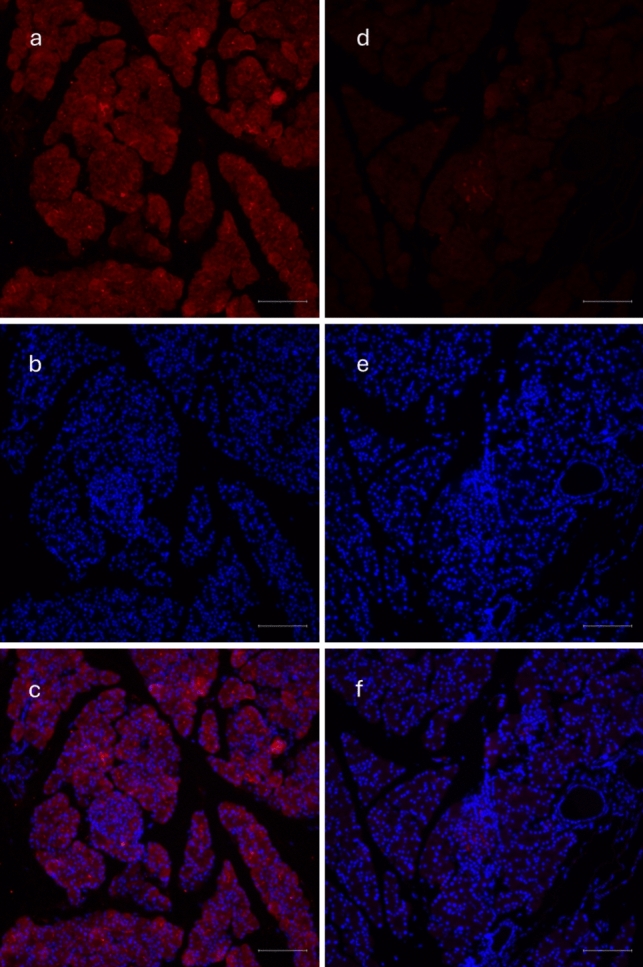
Immunofluorescence staining in rat pancreatic islet. **a** MOTS-c presence in rat pancreatic islet, **b** DAPI staining in rat pancreatic islet, **c** combination of** a** and** b**, **d** disappearing signal of MOTS-c, when using blocking peptide, **e** DAPI staining in rat pancreatic islet, **f** combination of** d** and** e**. Scale bar 100 µm

**Fig. 3:**
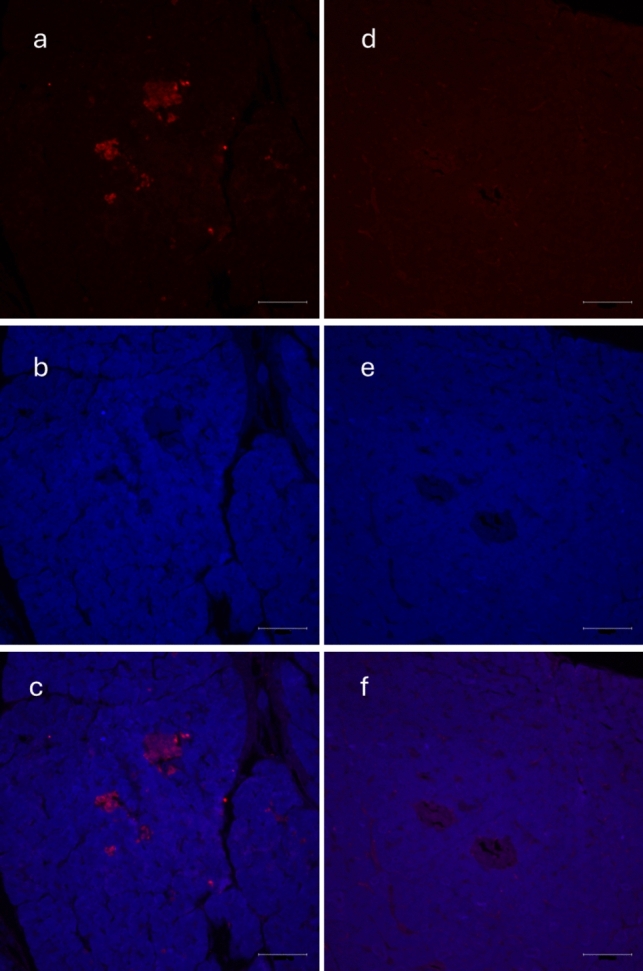
Immunofluorescence staining in rat pancreas: **a** MOTS-c, **b** insulin, **c** DAPI staining, **d** colocalization of MOTS-c and insulin in rat pancreas **e** MOTS-c, **f** glucagon, **g** DAPI staining, **h** colocalization of MOTS-c and glucagon in rat pancreas. Scale bar 100 µm

**Fig. 4:**
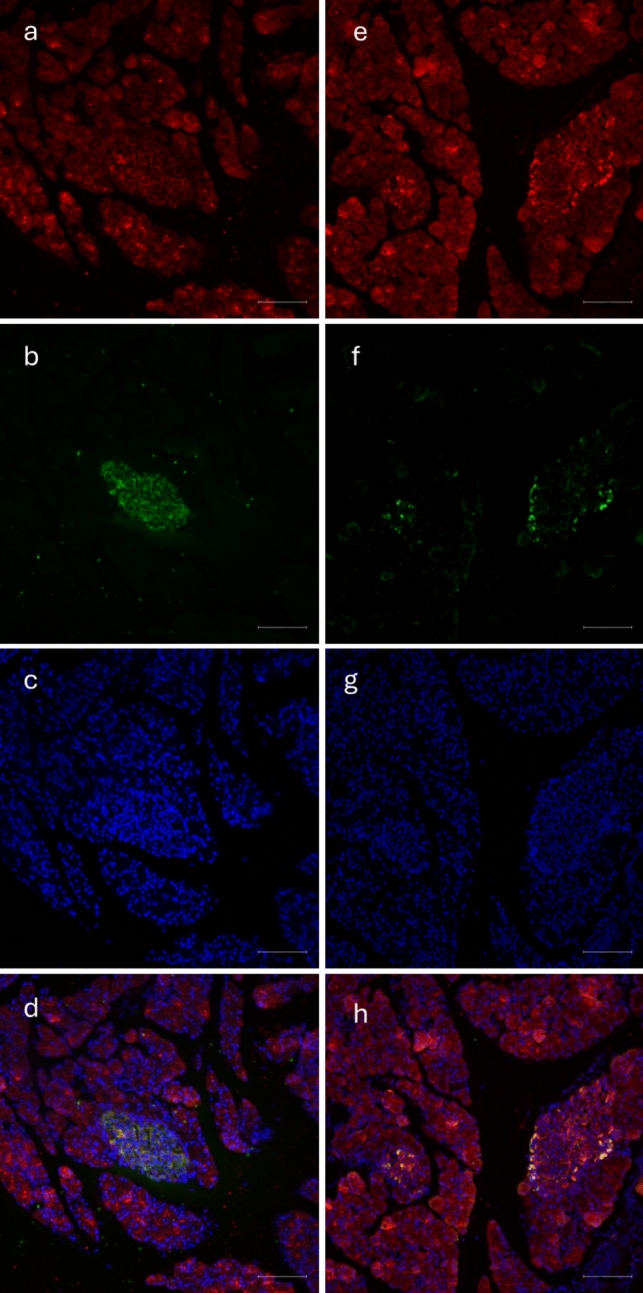
Immunofluorescence staining in pig pancreas: **a** presence of MOTS-c in pig pancreas, **b** DAPI staining, **c** combined pictures of** a** and** b**, **d** disappearing signal of MOTS-c after use of blocking peptide, **e** DAPI staining, **f** combination of** d** and** e**. Scale bar 100 µm

**Fig. 5:**
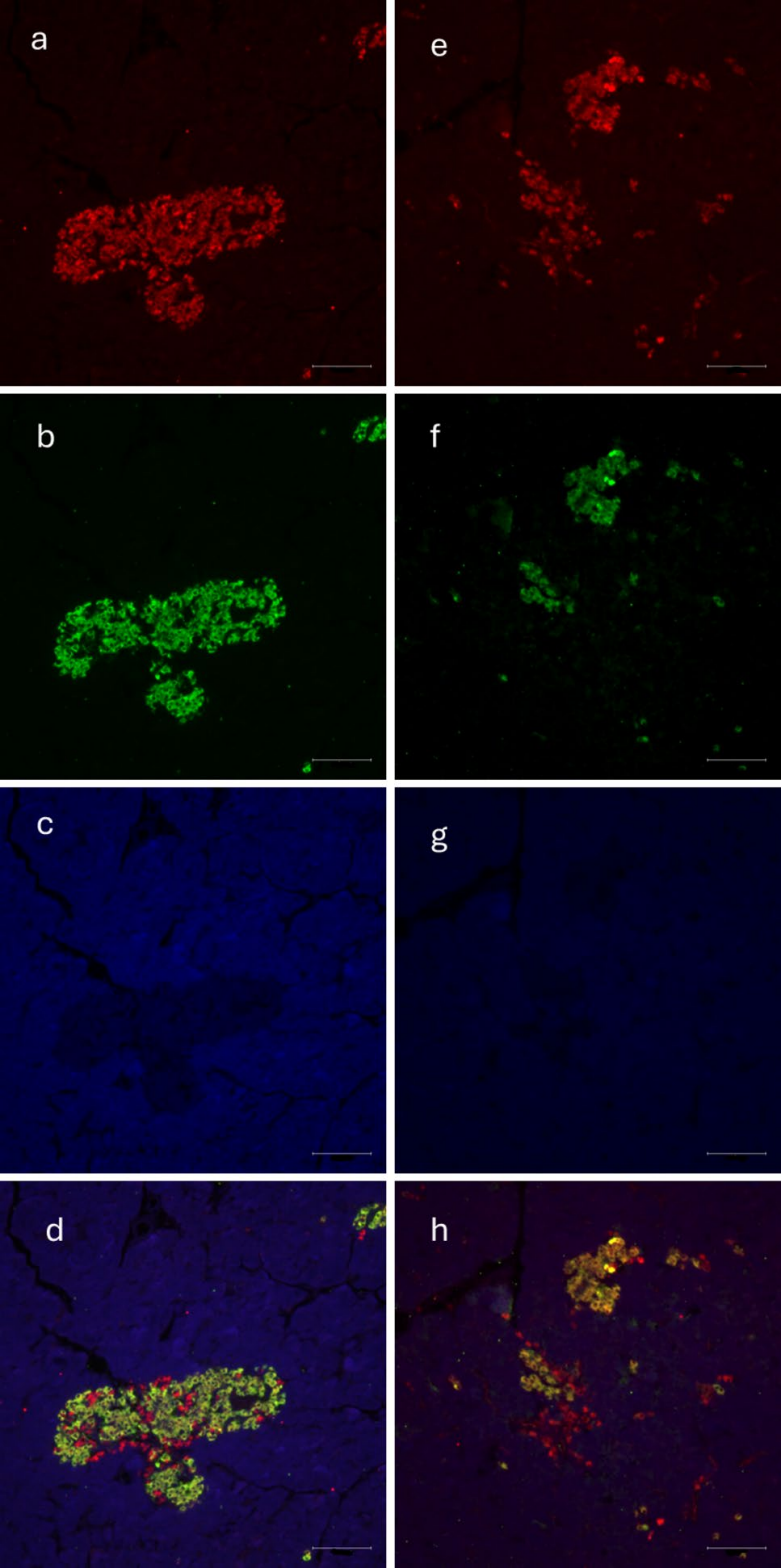
Immunofluorescence staining in pig pancreas: **a** MOTS-c, **b** insulin, **c** DAPI staining, **d** colocalization of MOTS-c and insulin in pig pancreas, **e** MOTS-c, **f** glucagon, **g** DAPI staining, **h** colocalization of MOTS-c and glucagon in pig pancreas. Scale bar 100 µm

Immunofluorescence staining of isolated pancreatic islets confirmed the presence of MOTS-c in both rat and pig islets (Figs. 2a and 4a). The specificity of this signal was eliminated using a blocking peptide (Figs. 2d and 4d). Additional staining for insulin and glucagon was conducted to assess the colocalization of MOTS-c with these hormones (Figs. 3d, h and 5d, h). In rats, MOTS-c was detected throughout the pancreas, including both endocrine and exocrine regions. In contrast, in pigs, MOTS-c expression was restricted to the islets, the endocrine portion of the organ. The detection of MOTS-c and insulin and glucagon in rats showed that MOTS-c is not produced by separate cells but colocalizes in cells, producing both insulin and glucagon. Colocalization is also observed in the pig, but in this species MOTS-c is produced by cells within the islets but not in all cells that also synthesize insulin or glucagon.

### Energetic compounds and pancreatic hormones change MOTS-c expression and secretion from rat pancreatic islets

**Fig. 6:**
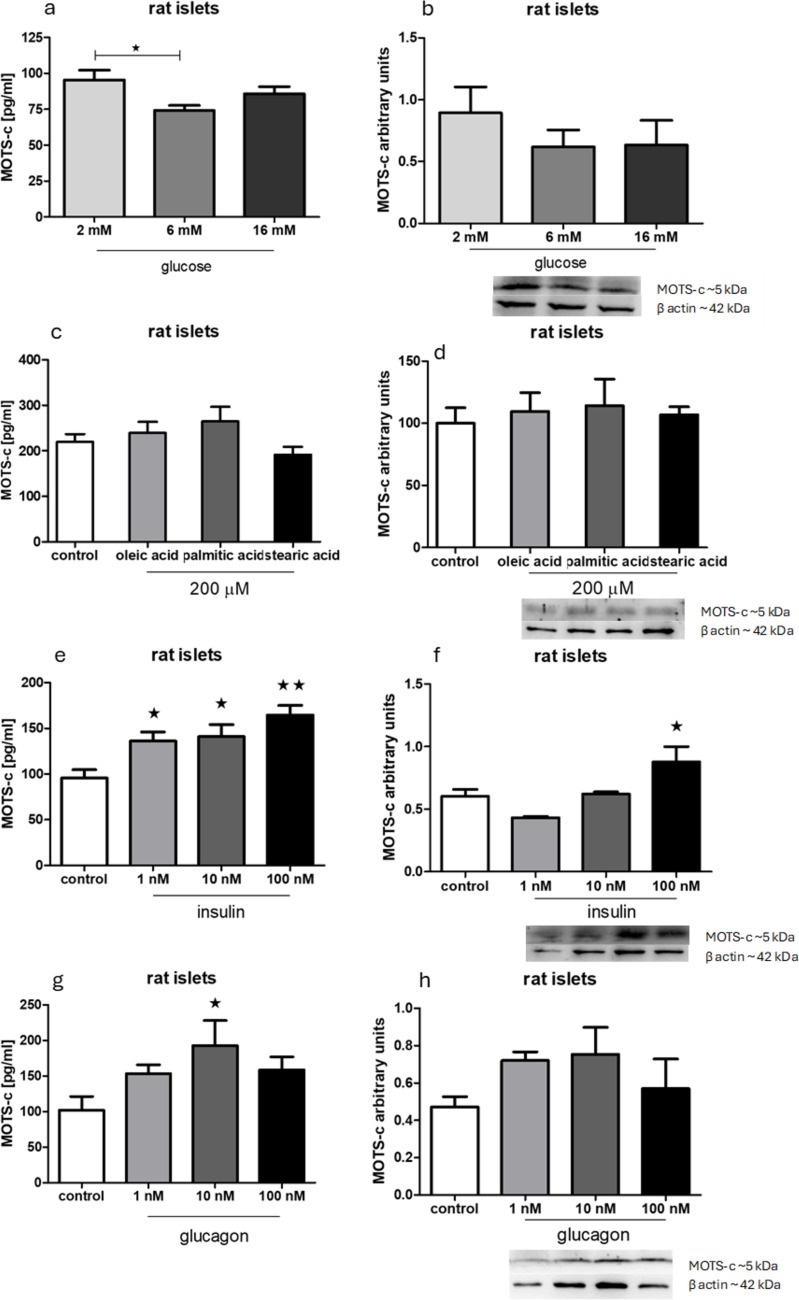
Influence of chosen energetic compounds and hormones on secretion and expression of MOTS-c examined with Western blotting in rat pancreatic islets. **a** MOTS-c secretion after incubation with different concentrations of glucose, **b** MOTS-c expression after incubation with different concentrations of glucose, **c** MOTS-c secretion after incubation with free fatty acids, **d** MOTS-c expression after incubation with free fatty acids, **e** MOTS-c secretion after incubation with insulin, **f** MOTS-c expression after incubation with insulin, **g** MOTS-c secretion after incubation with glucagon, **h** MOTS-c expression after incubation with glucagon. Data show mean ± SEM. *p < 0.05; **p < 0.01

A lower glucose concentration (2 mM) in the incubation buffer led to increased MOTS-c secretion, although no change was observed in protein expression levels in rat pancreatic islets (Fig. 6a, b). Incubation with free fatty acids in the medium did not produce significant changes in either MOTS-c secretion or expression (Fig. 6c, d). Significant changes were noticed after incubation of pancreatic islets with insulin: in each of the examined groups, there was significant enhancement of secretion of MOTS-c. Also, there was an increased expression of MOTS-c on protein level in islets treated with 100 nM insulin (Fig. 6e, f). Glucagon treatment induced a significant increase in MOTS-c secretion at the 10 nM concentration, but no change in protein expression was detected (Fig. 6g, h).

### MOTS-c lowers insulin and glucagon secretion but enhances its expression at the RNA level in rat pancreatic islets

**Fig. 7:**
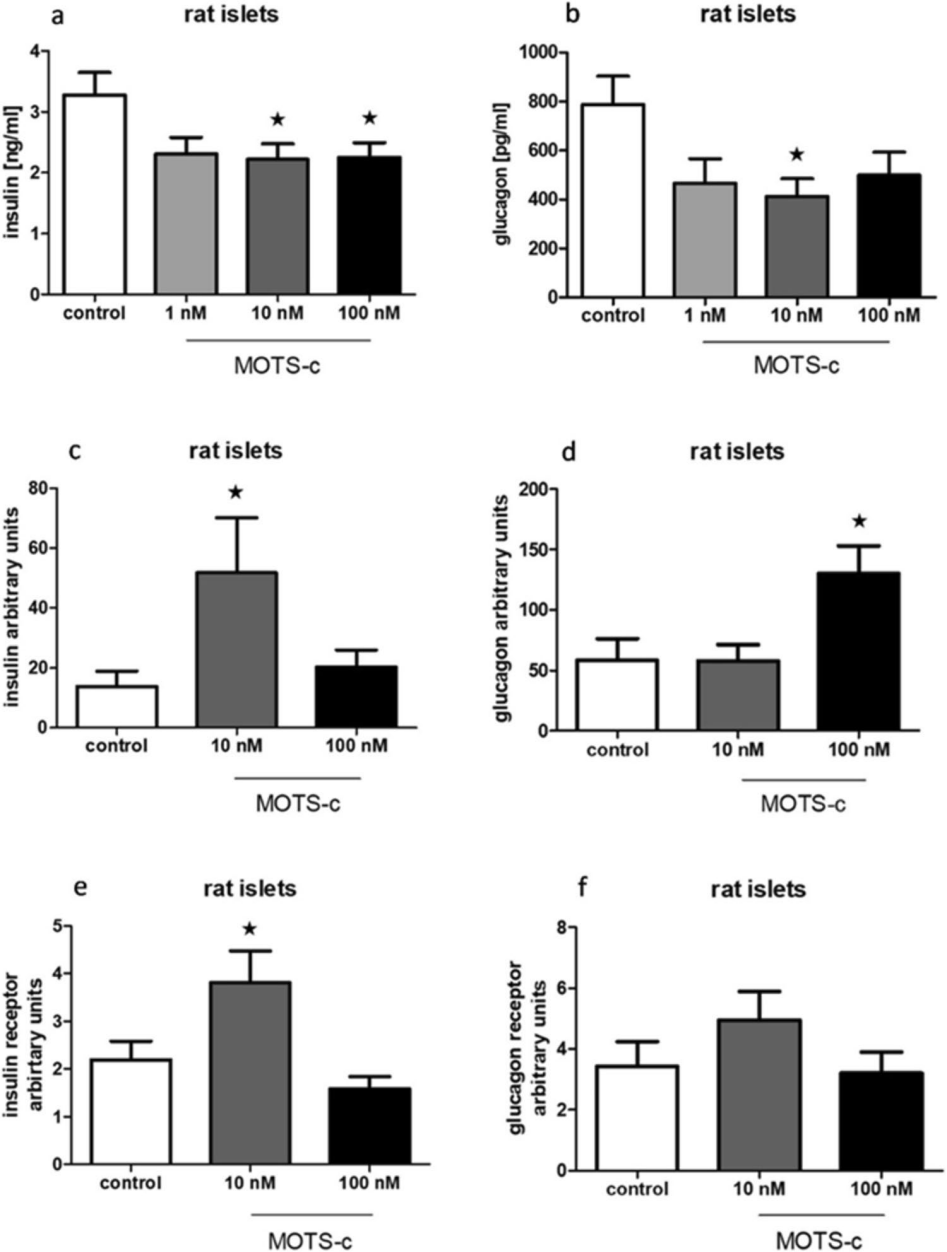
Influence of MOTS-c on secretion and expression of insulin and glucagon tested with PCR in rat pancreatic islets: **a** insulin secretion after incubation with MOTS-c, **b** glucagon secretion after incubation with MOTS-c, **c** insulin expression after incubation with MOTS-c, **d** glucagon expression after incubation with MOTS-c, **e** insulin receptor expression after incubation with MOTS-c, **f** glucagon receptor expression after incubation with MOTS-c. Data show mean ± SEM, *p < 0.05

Treatment of rat pancreatic islets with MOTS-c resulted in reduced insulin secretion at concentrations of 10 and 100 nM (Fig. 7a), and decreased glucagon secretion at 10 nM (Fig. 7b). The addition of MOTS-c to incubation medium enhanced insulin and glucagon expression (Fig. 7c, d). Tests were also performed measuring expression changes of insulin receptor and glucagon receptor after incubating rat pancreatic islets with different concentrations of MOTS-c; these showed increased expression on RNA level only in insulin expression in the group treated with 10 nM MOTS-c (Fig. 7e, f).

### Energetic compounds and pancreatic hormones change MOTS-c secretion but not MOTS-s expression on protein level in pig pancreatic islets

**Fig. 8:**
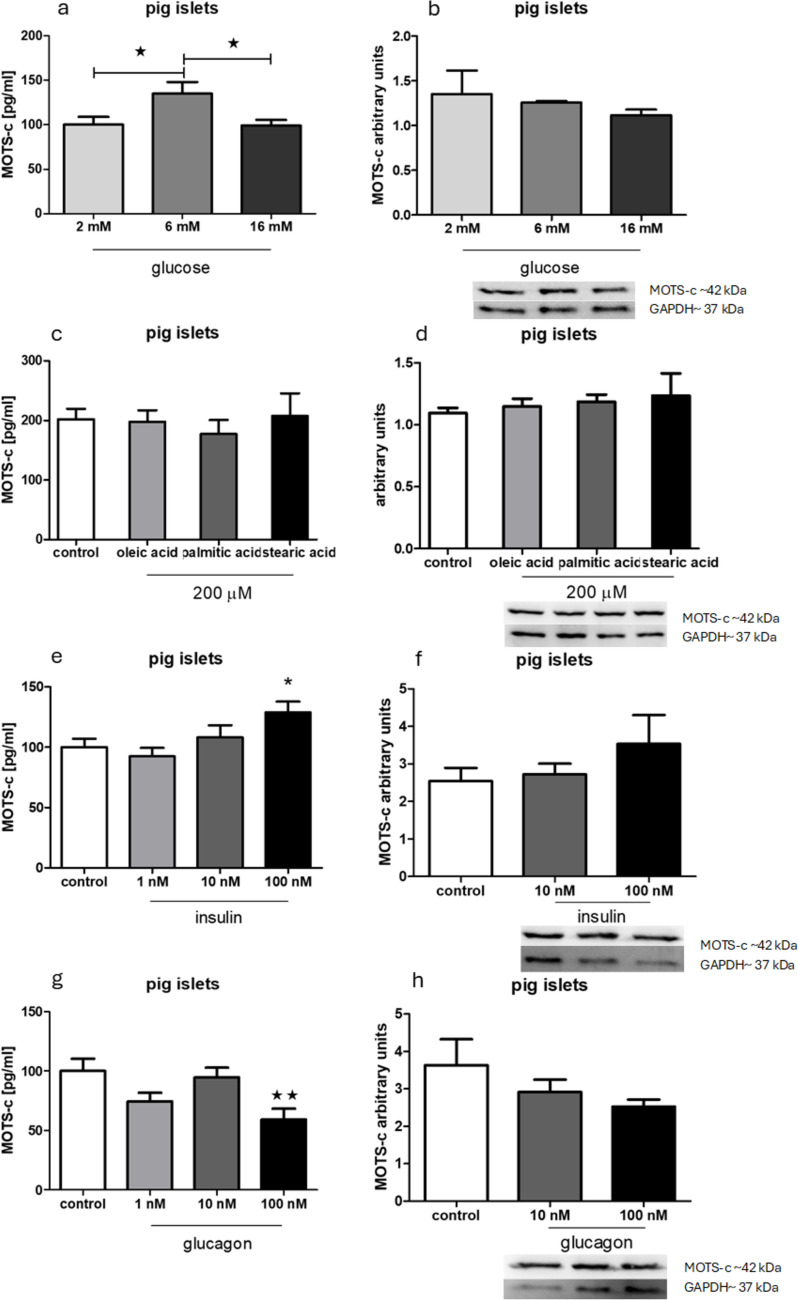
Influence of chosen energetic compounds and hormones on secretion and expression of MOTS-c examined with Western blotting in pig pancreatic islets. **a** MOTS-c secretion after incubation with different concentrations of glucose, **b** MOTS-c expression after incubation with different concentrations of glucose, **c** MOTS-c secretion after incubation with free fatty acids, **d** MOTS-c expression after incubation with free fatty acids, **e** MOTS-c secretion after incubation with insulin, **f** MOTS-c expression after incubation with insulin, **g** MOTS-c secretion after incubation with glucagon, **h** MOTS-c expression after incubation with glucagon. Data show mean ± SEM. *p < 0.05; **p < 0.01

Incubation of pig pancreatic islets in 2 and 16 mM glucose resulted in lower secretion of MOTS-c, but it did not change the expression of this peptide (Fig. 8a, b). Treating pancreatic islets with insulin and glucagon enhanced and diminished MOTS-c secretion in groups with 100 nM of each hormone, respectively (Fig. 8e, g). No significant changes in expression were observed after incubating pig pancreatic islets with those hormones (Fig. 8f, h).

### Insulin and glucagon lowers MOTS-c expression and enhances MOTS-c secretion in pig pancreatic islets

**Fig. 9:**
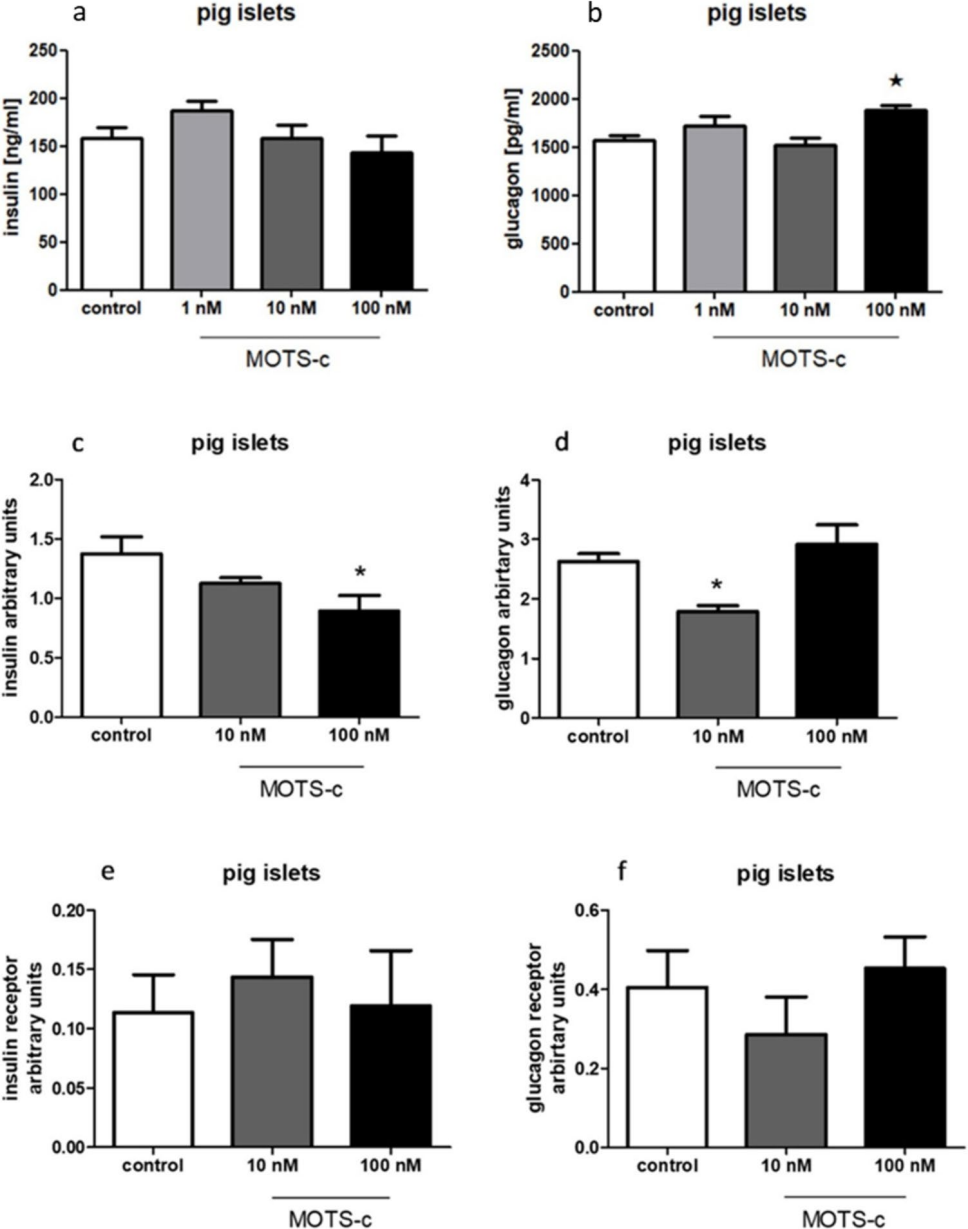
Influence of MOTS-c on secretion and expression of insulin and glucagon tested with PCR in rat pancreatic islets: **a** insulin secretion after incubation with MOTS-c, **b** glucagon secretion after incubation with MOTS-c, **c** insulin expression after incubation with MOTS-c, **d** glucagon expression after incubation with MOTS-c, **e** insulin receptor expression after incubation with MOTS-c, **f** glucagon receptor expression after incubation with MOTS-c. Data show mean ± SEM, *p < 0.05

A significant increase in the secretion of MOTS-c was observed in pig islets treated with 100 nM glucagon, whereas insulin treatment did not affect secretion (Fig. 9a, b). At the RNA level, MOTS-c expression was significantly reduced after treatment with 100 nM insulin and 10 nM glucagon (Fig. 9c, d). No changes were detected in the RNA expression of insulin or glucagon receptors (Fig. 9e, f).

### MOTS-c enhances the viability of pig and rat pancreatic islets but does not affect their apoptosis

**Fig. 10:**
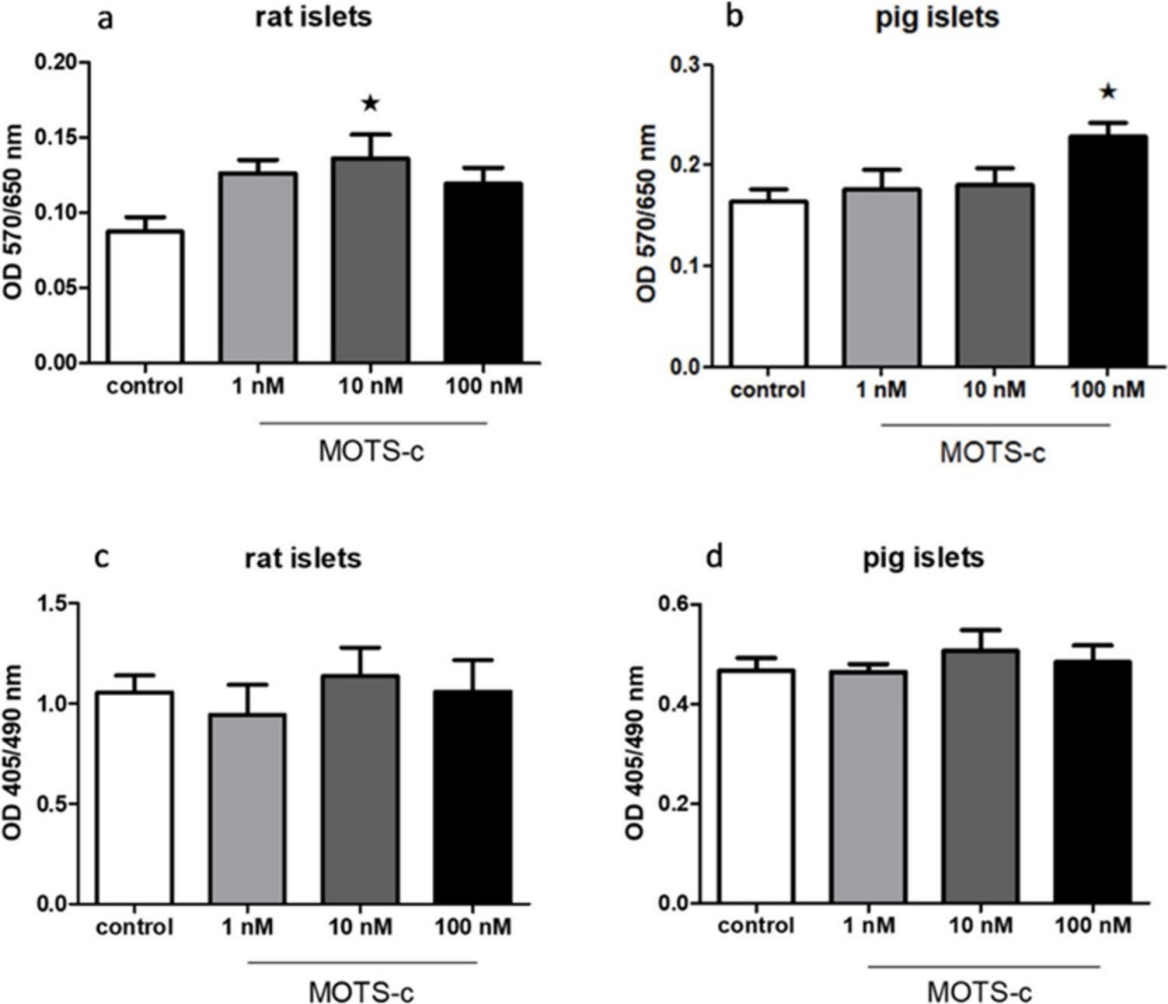
Influence of MOTS-c on pancreatic islet cells: **a** rat pancreatic islet cell viability measured with MTT test, **b** pig pancreatic islet cell viability measured with MTT test, **c** rat islet cell death measured with cell death test, **d** pig pancreatic islet cell death measured with cell death test. Data show mean ± SEM, *p < 0.05

Treating pancreatic islets obtained from pigs and rats with MOTS-c increased their viability (in groups with 10 and 100 nM, respectively; Fig. 10a, b). However, MOTS-c had no significant impact on apoptosis levels in either species (Fig. 10c, d).

## Discussion

In the context of metabolic disorders, it is well established that a Western diet and lifestyle contribute to the rising incidence of obesity and type 2 diabetes mellitus (Kopp 2019). One challenge for researchers is finding a way to prevent the spread of such diseases. New naturally occurring compounds continue to be discovered and evaluated for their physiological impact on both animals and humans. Our research team previously investigated MOTS-c, a peptide derived from the mitochondrial genome and discovered in 2015, which has been shown to enhance insulin sensitivity (Lee et al. 2015). Following promising results from experiments using cultured pancreatic cell lines (Bień et al. 2024), we extended our studies to more complex biological models. In selecting model species, our primary aim was to compare two morphologically distinct pancreatic types and assess whether MOTS-c modulates pancreatic islet function.

First, we confirmed the specificity of the antiMOTS-c antibody for the pig peptide. During this test, it was discovered that pig MOTS-c is larger than the corresponding peptide present in the rat pancreas. This is particularly important because the amino acid sequence of this peptide is still unknown (Lee et al. 2015). Moreover, since pig MOTS-c is a larger protein, it will be a fascinating subject for further experiments. As a result of the unavailability of a synthetic pig MOTS-c peptide and the physiological similarities between pigs and humans (Lunney 2021), we chose to use the human analogue in all pig-related experiments. Of course, we are aware of the limitations of this solution, but it was the only way to test the mentioned models. Since these two model animals have different pancreas anatomy, there were differences in the results of experiments as well. In rats, MOTS-c is present in the whole pancreas—in the endocrine and exocrine parts. In pigs, MOTS-c occurs only in the endocrine part of the pancreas (Figs. 1, 2, 3, and 4). Together with the different sizes of the porcine peptide, this may indicate significant differences in the physiological role of MOTS-c between these species. As mentioned before, the pig is an excellent model for humans, and its pancreas is anatomically closer to humans than the rat one; we can assume that the results obtained on the swine model could be more related to what is expected in humans. Rodents are also good laboratory animals because they are relatively cheap and easy to care for. They are good sources of information on how newly discovered peptides work, but sometimes the functions of analogous compounds in humans may differ significantly (Arner 2005).

MOTS-c is derived from mitochondria (Zhong et al. 2022). Therefore, we performed experiments testing the influence of energetic compounds such as glucose and free fatty acids on MOTS-c secretion and expression. In addition, we examined the effects of pancreatic hormones on MOTS-c secretion in both rat and pig islets. It is important to note that results related to MOTS-c, insulin, or glucagon—whether regarding secretion or expression—may not directly correlate. This discrepancy is largely due to differences in the duration of experimental procedures: secretion experiments lasted 1.5 h, whereas expression analyses via PCR or Western blot required 24 h. The results from rat islet experiments were consistent with those obtained in previous studies using cell lines (Bień et al. 2024), while the findings from pig islets did not show similar patterns. This may be attributed to the rodent origin of the INS-1E and αTC-1 cell lines used in earlier studies. However, not all outcomes aligned across models. For instance, the effects of free fatty acids on MOTS-c secretion observed in cell lines were not replicated in the more complex islet models (Figs. 6c, d and 8c, d). Therefore, experiments should be performed on laboratory cultured cell lines since they have limitations and on larger structures like islets or in vivo tests.

Another observation supporting the hypothesis that MOTS-c functions differently in rats and pigs is the contrasting regulation of its secretion by glucose (Figs. 6a and 8a). In rats, MOTS-c secretion is lowest at physiological glucose concentrations, whereas in pigs, it is highest. This suggests distinct regulatory roles for MOTS-c in these species. However, this is only speculation because of the lack of the porcine MOTS-c sequence and the lack of known receptors for this peptide.

In rat pancreatic islets, we observed a feedback loop consistent with our earlier findings in INS-1E cells. Specifically, MOTS-c reduces insulin secretion, while insulin enhances MOTS-c secretion (Figs. 6e and 7a). A similar feedback pattern was noted with glucagon secretion (Figs. 6g and 7b).

Regarding pig islets, we see no similar feedback loops with insulin and glucagon, as no correlations were observed (Figs. 8e, g and 9a, b). Other results obtained on rat pancreatic islets and cell lines showed increased insulin receptor expression in rat pancreatic islets and cell lines, but this effect was not observed in pig islets. This discrepancy may help explain the mechanism by which MOTS-c enhances insulin sensitivity in rats—a well-documented property of the peptide (Lee et al. 2015; Kim et al. 2019).

The final stage of this study examined whether MOTS-c influences cell viability and apoptosis. Previous studies have shown that this peptide improves muscle function in mice (Ran et al. 2021) and has demonstrated protective effects in pancreatic cell lines (Bień et al. 2024). Although MOTS-c did not affect apoptosis levels in pancreatic islets from either species, it significantly enhanced cell viability in both pig and rat models. This finding may be especially relevant for extending the functional lifespan of pancreatic islet cells during the progression of diabetes, a protective role that has already been confirmed in rodent models of type 1 diabetes (Kong et al. 2021).

In summary, MOTS-c is a mitochondria-derived peptide intimately linked to metabolic regulation. Although research into its mechanisms and effects is ongoing, many of its physiological roles remain unknown—most notably, the receptor to which it may bind has yet to be identified. Our results suggest that MOTS-c differs fundamentally between rats and pigs, both structurally and functionally: (i) the sizes of MOTS-c of rats and pigs are different; (ii) the locations in the rat and pig pancreas are different; (iii) its secretion patterns in response to glucose and glucagon differ; and (iv) the regulatory effects of insulin and glucagon on MOTS-c secretion in rats and pigs are different. Such a discrepancy in animal data may be more worrying than the widespread availability of MOTS-c as a supplement for people, which is treated almost as a substitute for physical exercise. It is therefore not surprising that the World Anti-Doping Agency (WADA) has included MOTS-c on its list of banned substances in sports (WADA 2025).

## Data Availability

No datasets were generated or analysed during the current study.
